# A roadmap for improving data quality through standards for collaborative intelligence in human-robot applications

**DOI:** 10.3389/frobt.2024.1434351

**Published:** 2024-12-12

**Authors:** Shakra Mehak, Inês F. Ramos, Keerthi Sagar, Aswin Ramasubramanian, John D. Kelleher, Michael Guilfoyle, Gabriele Gianini, Ernesto Damiani, Maria Chiara Leva

**Affiliations:** ^1^ Pilz Ireland Industrial Automation, Cork, Ireland; ^2^ School of Food Science and Environmental Health, Technological University Dublin, Dublin, Ireland; ^3^ Secure Service-oriented Architectures Research Lab, Department of Computer Science, Università degli Studi di Milano, Milan, Italy; ^4^ Robotics and Automation Group, Irish Manufacturing Research Centre, Mullingar, Ireland; ^5^ School of Computer Science and Statistics, Trinity College, Dublin, Ireland; ^6^ Department of Informatics, Systems and Communication (DISCo) Università degli Studi di Milano-Bicocca, Milano, Italy

**Keywords:** human robot interaction (HRI), collaborative intelligence, ISO standard, human machine interaction, artificial intelligence, machine learning, ISO 8000

## Abstract

Collaborative intelligence (CI) involves human-machine interactions and is deemed safety-critical because their reliable interactions are crucial in preventing severe injuries and environmental damage. As these applications become increasingly data-driven, the reliability of CI applications depends on the quality of data, shaping the system’s ability to interpret and respond in diverse and often unpredictable environments. In this regard, it is important to adhere to data quality standards and guidelines, thus facilitating the advancement of these collaborative systems in industry. This study presents the challenges of data quality in CI applications within industrial environments, with two use cases that focus on the collection of data in Human-Robot Interaction (HRI). The first use case involves a framework for quantifying human and robot performance within the context of naturalistic robot learning, wherein humans teach robots using intuitive programming methods within the domain of HRI. The second use case presents real-time user state monitoring for adaptive multi-modal teleoperation, that allows for a dynamic adaptation of the system’s interface, interaction modality and automation level based on user needs. The article proposes a hybrid standardization derived from established data quality-related ISO standards and addresses the unique challenges associated with multi-modal HRI data acquisition. The use cases presented in this study were carried out as part of an EU-funded project, Collaborative Intelligence for Safety-Critical Systems (CISC).

## 1 Introduction

The emergence of CI with Industry 4.0 represents a significant convergence between human and Artificial Intelligence (AI) abilities, becoming one of the main focus of the transition to Industry 5.0 Demir et al. (2019). Particularly in the field of collaborative robotics, where humans, robots and AI work in collaboration alongside each other, leading to a new age of enhanced productivity, adaptability and innovation Villani et al. (2018). The term CI refers to the execution of shared tasks by humans and AI systems in which they exploit the strengths of each other to increase productivity Toniolo et al. (2023). This concept is often studied under different terminologies, human-centric AI Rožanec et al. (2023), human-machine-in-loop Mosqueira-Rey et al. (2023) and human-AI interaction Amershi et al. (2019). However, the current immaturity of certain capabilities of AI, such as lack of intuitive reasoning, creativity, common sense, and situational analysis, as well as limited capabilities of humans in tasks that require long-term memory, repetition, speed, and accuracy, restrict the application of either full automation or solely manual operations to complex production environments (Villani et al., 2018). Furthermore, collaboration enables flexible and adaptive access to system resources, resulting in a more advantageous solution than swapping between human and machine tasks (Angleraud et al., 2021; Zhou et al., 2022).

One of the fundamental applications of the CI system is Human-Robot Interaction (HRI), which allows humans and robots to work together as a team (Panagou et al., 2024) and complement each other’s skills. The implementation of HRI systems necessitates the integration of heterogeneous data, including various data sources from humans and robots. This includes data on robot dynamics and kinematics, system and process state data from internal repositories, workspace and environment data from extrinsic sensors, human-related data from wearable and other sensors, human feedback from controllers or interfaces, and intelligent predictive or prescriptive information from an AI component. Moreover, these systems rely on real-time sensor data from the environment and human activity to recognize changes in the environment, enabling the customization of robot behaviour in accordance with operator preferences (Heinisch et al., 2024; Gao et al., 2021; Gast et al., 2009). To build a smooth interaction between humans and robots, a high quality multimodal data is required to be processed to design and develop a synergistic HRI system. There is comprehensive evidence demonstrating the correlation between data quality and AI model performance in the literature. However, data quality’s role in CI system development and performance is a less explored area but equally critical in collaborative systems because of the interactive nature of CI (Amershi et al., 2019; Rožanec et al., 2023; Gudivada et al., 2017). The main attributes of CI systems are accurate and timely decision-making (Mentzas et al., 2021), transparency (Vössing et al., 2022), user trust (McGrath et al., 2024) and poor data quality may result in mismatched in the human-machine interactions and inaccurate and untrustworthy system behaviours. Empirical studies on CI systems show that data quality directly affects system performance (Li et al., 2023), however, the extent of data quality’s consequence on CI systems remains an open ground for further research.

Data quality has been thoroughly explored beyond the HRI fields and has gained significant attention from the industry due to the multifaceted value that data can bring to the business (Batini et al., 2009). This effort can be observed by the development of data and data quality-related standards, namely ISO/IEC 25000 series (SQuaRE) (ISO/IEC 25000, 2014a), which describes requirements and evaluations for the quality of systems and software, and (ISO 8000-61, 2022a), which details data management processes. Despite this, the lack of standards practice and inadequacy in data-related standards (Odong et al., 2022) is a substantial constraint in achieving reliable CI systems. In this regard, this study aims to contribute to the literature by providing a simple framework for the standardization of data processes and data quality in HRI.

This article contributes as follows.

• The paper describes the key data quality challenges specific to multimodal HRI data acquisition for CI application, dissecting the complexities of data collection from various sensors and input types in industrial scenarios, also considering data obtained from interactions between humans and robots as well as, metrics related to physiological measures indicative of human cognitive and physical state, and subjective responses.
• Review of existing ISO standards and guidelines specifically addressing data quality for multimodal HRI data acquisition and the gaps identified for their applicability and their capacity to cover all data quality issues in multimodal HRI data.
• Hybrid standardization approach and a five-step data quality management plan based on ISO standards that can be effectively adapted for CI applications.
• Demonstration of the application and efficacy of the proposed hybrid standardization approach in managing data quality within two specific case studies in industrial HRI.

The paper is structured as follows: Section 2, provides a comprehensive background, which introduces collaborative intelligence, human-robot interaction, and an overview of data quality standards relevant for it. Section 3 presents a hybrid standardization for CI. Section 4 discusses two HRI use cases, and presents them to highlight the data collection methodology applied to the case studies and the issues related to data quality compared to the “Data Management Steps for CI applications” proposed in section 3. Section 5 summarizes the contributions, discusses their implications, and proposes potential avenues for future research.

## 2 Background and literature review

### 2.1 Collaborative intelligence (CI)

As previously discussed, CI is the seamless integration of human cognitive abilities with AI systems in order to facilitate decision-making, problem solving, and development in many different areas. Unlike conventional automation paradigms that aim to replace human work with machines, CI promotes collaboration between humans and AI systems. This approach takes advantage of the unique strengths of both entities to produce results that neither could independently achieve (Dellermann et al., 2021; Schleiger et al., 2024).

The implications of CI are disruptive in industries, as it improves efficiency and safety. For example, CI systems merge human insights with AI to optimize production demands, streamline the process, and reduce production time (Trakadas et al., 2020) in the manufacturing sector. Furthermore, it improves workplace safety by integrating human supervision with AI-powered surveillance technologies to monitor and predict safety incidents (Zheng et al., 2023).

The evolution of CI has been accompanied by important technological developments, particularly in the domains of machine learning (Moustakis and Herrmann, 1997; Semeraro et al., 2023), data analytics (Nardo et al., 2020), and Internet of Things (Bissoli et al., 2019). Current advances in the field of CI emphasize the necessity of adaptive learning (Sharifi et al., 2021) that can improve system responsiveness based on user feedback (Mehta and Losey, 2023), supporting sophisticated and contextually sensitive collaboration between human and AI systems. Moreover, CI in an industrial setting faces a plethora of challenges, starting with the need for heterogeneous data from multiple sources (Victorelli et al., 2020) subjective and contextual user feedback, the privacy and confidentiality of data streams (Karat et al., 2007) and data quality management. Addressing these challenges is critical if one wants to exploit CI systems to their full potential. This study focuses primarily on the quality of the data, which serves as the foundation for successful human-AI collaboration.

### 2.2 Data quality

The role of data quality cannot be overstated in many fields. Several works have been published on data quality challenges and management in AI models, machine learning (ML), and Internet of Things systems. Studies by (Ehrlinger and Wöß, 2022; Gong et al., 2023; Zhou et al., 2021) highlight the challenges in achieving high-quality data in ML, pointing out that the complexity of data sources often outpace current standardization efforts, such as those proposed by (ISO/IEC 25012, 2008a). Recently, (Eckman et al., 2024) has introduced the application of survey methodologies to improve data collection processes, suggesting that the strategies developed for survey data quality can be adapted to improve ML data acquisition. By examining data quality aspects within ML pipelines, including data collection, pre-processing, and validation, (Rangineni, 2023) emphasize the imperative of adhering to quality standards in ML development pipelines, exploring the direct correlation between data quality and model performance. Furthermore, (Gudivada et al., 2017) proposed a data quality management framework for big data and ML, highlighting the importance of standards to ensure data integrity in a large-scale data environment. The authors in (Gupta et al., 2021) discuss the lack of focused efforts to improve data quality in current research practices and underscore the fundamental limitation that the performance of ML models is bounded by the quality of the data used for training. Another work by (Perez-Castillo et al., 2018) proposed a (ISO 8000-61, 2022a) based quality management framework for sensor data to overcome the challenges associated with data quality activities in smart systems. Moreover, the authors in (An et al., 2020) investigate the application of blockchain technologies as a method to improve data integrity and security in IoT systems. Their work presents a decentralized approach to data quality management, using blockchain’s inherent properties to ensure reliability of IoT data.

Collectively, these studies highlight the importance of data and dataset quality in system development and functionality, and advocate for a multidisciplinary approach, combining insights from data science, ML and quality management to develop strategies to ensure data reliability in complex computational environments.

### 2.3 Human robot interaction and data quality challenges

The field of HRI is significant within the broader domain of collaborative intelligence. It is dedicated to understanding, developing, and refining the interfaces and interactions between humans and robotic systems, ensuring that such collaborations are as effective and efficient as possible. The type of interaction includes both direct physical interaction and shared decision-making processes, in which both humans and robots contribute their respective abilities. Moreover, HRI exceeds traditional automated systems, introducing robots capable of working alongside human operators in shared spaces (Hentout et al., 2019).

The development of human data-driven HRI systems has resulted in notable advances in productivity and safety within the sector (Liu et al., 2016; Saheb Jam et al., 2021; Admoni and Scassellati, 2014; Ravishankar et al., 2024; Yang et al., 2021). In addition, the utilization of human feedback insights such as cognitive or emotional state data (Khamassi et al., 2018), visual data (Gao et al., 2021), and direct communication cues (Khamassi et al., 2018) enable HRI systems to adapt and respond to the needs and safety of human operators. In addition, these data can be used in the training of adaptive models to allow robots to adjust their behaviors and functionality to improve collaboration (Skantze et al., 2014). Through understanding of the stress levels, concentration, and fatigue experienced by human operators, robots possess the ability to independently adapt to or notify humans of potential hazards, creating a working environment that is reliable and responsive (Bekele and Sarkar, 2014). Furthermore, the successful implementation of HRI is based on the precision, dependability, and timeliness of the data used in the development and training of the model.

In HRI systems the quality of the data depends on the purpose of their use, however, common data quality challenges include: (a) the operationalization of relevant human signals or feedback (Kim et al., 2020), (b) the distinction between noise, confounding effects, and individual variation, (c) the prevention or correction of missing data due to sensor malfunction (Teh et al., 2020), recording or storing issues, loss of synchronization or human agent non-compliance with the data collection process (Celiktutan et al., 2017), or (d) the capture of relevant environmental, workplace and task contexts that impact the operational design domain and ecological validity of the system functions (Hoffman and Zhao, 2020; Han and Williams, 2022). In this context, the impact of data quality degradation can be measured by its impact on HRI quality, as assessed by different robot-related metrics, human-related metrics or human-robot interaction-related metrics (George Kokotinis et al., 2023).

### 2.4 An overview of ISO standards regarding data quality

ISO standards explicitly referring to data quality are significant within industrial systems, as they establish a structure for evaluating, controlling, and improving data quality throughout its entire lifetime. These standards set forth criteria and guidelines to ensure that data, regardless of their origin, meet the strict criteria necessary for precise analysis and decision-making. In the context of CI systems, where decisions are based on data-driven AI analysis and human input, adherence to these standards is imperative for CI data. Table 1 summarizes key international data standards that present data quality frameworks and management protocols that can be suitable for CI data. To demonstrate the importance of data quality standards for collaborative data, we have also specified their impact level based on their contribution to the foundational quality (Perez-Castillo et al., 2018; Batini et al., 2009; Panduwiyasa et al., 2021) and expert opinion. For instance, ISO 8000-61 (ISO 8000-61, 2022a) is marked as high due to its critical role in establishing the data quality framework, and it is widely adopted and highly endorsed for its comprehensive approach to data quality (Perez-Castillo et al., 2018).

**TABLE 1:** Summary of the key data quality standards for HRI systems.

Standard	Title	General description	Relevance to data quality	Relevance to CI/HRI context	Impact
IEC 8000-61 ISO (2022a)	Data Quality Management: Process reference modal	Provides a process reference model framework for data quality management, including principles for data quality planning and control	Fundamental to measure the quality of collected data and improves the processes to avoid data nonconformity	Suitable for managing heterogeneous data sources and identifying potential risks associated with data or system	High
ISO/IEC 25010 ISO (2023a)	System and Software Engineering	Defines software product quality model requirement elicitation and specification	Integral to quality testing and quality assurance	This can be applied to define CI data quality in use, regarding the effectiveness, productivity, safety, and satisfaction of the user interaction with the system	Medium
ISO/IEC 25022 ISO (2008b)	System and Software Engineering	Defines quality in use, intended to be use with other software quality related standards	Provides evaluation process and normalizing quality in use assessment	Offers similar role like ISO (2023a)	Low

Standard	Title	General Description	Relevance to Data Quality	Applicability to CI/HRI Context	Significance
ISO/IEC 25012 ISO (2008a)	Software Engineering	Defines a quality model for data used in software systems, covering 15 data quality characteristics	Address data quality characteristics	The quality characteristics can be used for data quality assessment of captured user feedback, interaction experience and data interpretations	High
ISO/IEC 25024 ISO (2015b)	System and Software Engineering	The standard provides set of specific quality measures for each related components and corresponding entity	Defines quantitative measures to assess data quality and describes an explanation of applying those measures for quality assessment	The quantitative measures can be used to analyze patterns and correlations of collected multimodal data for the implementation and development of the HRI system	Medium
ISO/IEC 25030 ISO (2019)	Software Engineering Quality Requirements	Focuses on the requirements for software product or data quality, use for requirement definition, use and govern	The offered framework in this standard use to evaluate that the data meets the defined and expected quality criteria	Supports in determining clear contexts in which the CI system will operate and ensuring optimal functionality in different conditions	Low
ISO/IEC 23053 ISO (2022c)	Framework for AI System Using ML	Offers data acquisition and data preparation steps in the ML pipeline	Pertinent for the development and maintenance of AI components in CI systems	The standard supports the development, deployment, and management of AI systems within collaborative environments, ensuring that these systems are built and operated transparently and effectively	High

Among these, the ISO 8000 series plays a crucial role in the context of data quality management by providing a comprehensive set of standards. These standards are designed to effectively monitor data quality in various business operations. Specifically (ISO 8000-1, 2022b), emphasizes the importance of data quality in achieving specific goals and objectives. The standard promotes a structured approach to data quality and governance to improve the decision-making process. According to this standard, data quality is described as an objective concept that can be assessed based on how well the data aligns with its intended purpose. By offering a wide-ranging framework, (ISO 8000-1, 2022b) addresses data quality across different data types, formats, and organizational settings. It can also be applied to leverage data in operational activities and model training for ML systems, which can be relevant for CI systems. However, the standard needs more detailed information on the specific processing methods involved. Furthermore (ISO 8000-8, 2015a), defines and measures the quality of information and data quality based on three categories: *syntactic*, *semantic* and *pragmatic* quality. *Syntactic* quality measures conformance to syntax requirements, *semantic* quality measures data alignment to what it represents in the system, and *pragmatic* quality measures the suitability of the data for a particular application. For CI data, semantic quality verification can involve, (a) Identifying any missing data due to sensor malfunction, (b) data acquisition issues, (c) loss of synchronization or human agent non-compliance with the data collection process, and (d) ensuring that data variances match expected patterns to appropriately map data to entities in the operational domain (ISO 8000-8, 2015a). The practical relevance of data in CI contexts depends on its *pragmatic* quality, which considers the usefulness of the data against costs and benefits, particularly important when using hard-to-collect human data. The ISO 8000-61 (ISO 8000-61, 2022a) presents a structured data quality management approach. The reference model begins with comprehensive data quality planning, requirement specifications, rigorous data processing, monitoring, and management. The framework for considering the characteristics of the CI data in the present study is specifically derived from the ISO 8000-61 (ISO 8000-61, 2022a).

The ISO 25000 (ISO/IEC 25000, 2014a) series, addresses the Systems and Software Quality Requirements and Evaluation (SQuaRE). They offer a comprehensive framework to guarantee the quality of software products and systems, mainly those relevant to ML and potentially CI systems. The key standards of this series include (ISO/IEC 25001, 2014b), which handles quality management; ISO 25012 (ISO/IEC 25012, 2008a), which focuses on the data quality model; and (ISO/IEC 25030, 2019), which deals with quality requirements. ISO 25012 (ISO/IEC 25012, 2008a) defines a data quality model required to assess data quality needs across multiple data processes such as production, collection, and integration, emphasizing the importance of data quality within compliance guidelines. It covers different data quality attributes needed to maintain the integrity and practicality of data within machines and human interaction. These factors, such as correctness, completeness, consistency, credibility, and efficiency, are essential to ensure the reliability and effectiveness of the data (ISO 8000-61, 2022a). As human data is commonly used within the scope of CI, the concepts of compliance, efficiency, traceability, and accessibility are significant for human factor impact assessment Schleiger et al. (2024). In addition (ISO/IEC 25010, 2023a), introduces a quality model for software that extends beyond data to incorporate the quality of use, focusing on quality assurance and used in conjunction with (ISO/IEC 25012, 2008b). To achieve optimal results, it is critical to take into account both the data and the application environment, as this perspective is fundamental to CI applications.

ISO/IEC 22989 (2023b) establishes a framework for AI through the definition of key concepts and terminology that encompass various aspects of AI, such as systems and data. It underscores the importance of data precision, the process of training and validating ML models and the iterative nature of retraining models, and highlights the ongoing evolution of AI systems to adapt and improve themselves over time. Furthermore, the standard emphasizes the critical role of data quality, annotation, augmentation, and the significance of data sets in the training, validation, and testing of AI models, thus influencing the efficacy and reliability of AI systems. The lifecycle of an AI system comprises multiple stages, with the standard stressing the pivotal role of data at each phase, for example, training, validation, and testing. These stages are fundamental in the evolution of AI models, necessitating the use of high-quality, varied, and inclusive datasets to construct models that function dependably and impartially in real-world settings. By offering a vocabulary for discussing the AI system’s lifecycle, the standard facilitates the integration of AI into collaborative environments, ultimately supporting the adoption of CI systems. Similarly (ISO/IEC 23053, 2022c), offers a systematic framework for data management in ML systems. This standard focuses on the key stages of data collection, preprocessing, and validation to ensure the efficiency and dependability of AI models. The procedure highlights the importance of precise data classification by segregating training, validation, testing, and production data sets while ensuring statistical uniformity to enhance model evaluation and practicality. The standard emphasizes the processes of cleaning and normalizing the input-output data arrangements to enhance the performance of the model. It is important to note that complying with the systematic methodology for data management outlined in (ISO/IEC 23053, 2022c) is a prerequisite for developing AI systems that demonstrate the ability, dependability, and adaptability required for certain application domains.

## 3 Hybrid standardization

Hybrid standardization refers to a systematic approach to identifying related standards that combines the strengths of system-driven selection and collaborative preference among stakeholders (Van Wegberg, 2004). It allows the re-contextualization of several standards to better cover the emerging needs of complex systems with multiple interacting components.

**FIGURE 1:**
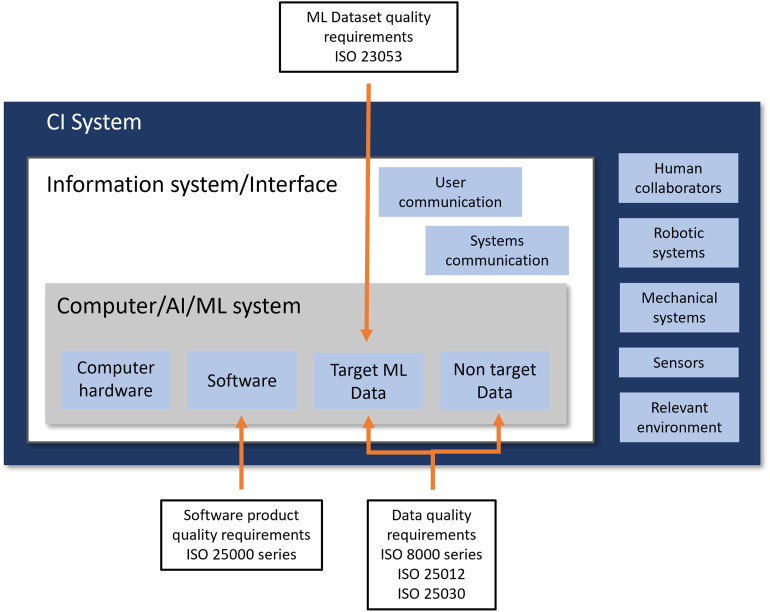
Hybrid standardization framework for CI (system) data quality management.

This study proposes a hybrid standardization framework to improve data quality, specifically in CI systems. A CI system, as depicted in Figure 1, is composed of several subsystems, including the Computer/AI/ML subsystem(s) that stores, manages, and employs both “target ML data” and “non target data,” distinguishing the type of data. The information and interface subsystem is responsible for user communication and between-systems communication functions which is an essential component of CI systems. Other subsystems include human collaborators, robotic or mechanical subsystems, sensor subsystems, and environmental components. Data streams from any of these sub-components, often from more than one, can be used by the ML/AI component or as contextual supportive information to implement the required CI functions.

**FIGURE 2:**
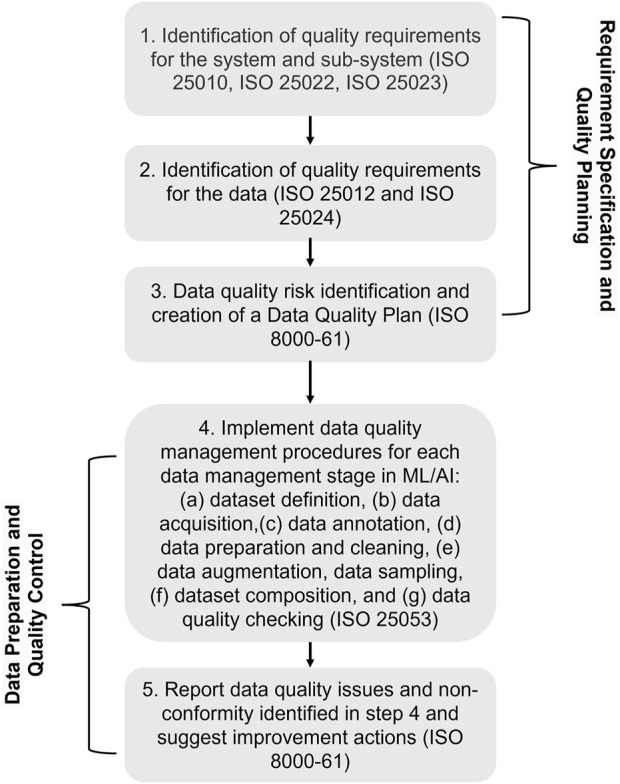
Data Management Steps for CI applications.

The framework takes into account the changing nature of CI data, which involves complex interactions with human-generated content that is subjective and context-dependent. Moreover, it uses current data standards with best practices in ML data management and identifies and applies specific standards to two main data categories within the CI system. They are “target ML data” and “non-target data.” For “target ML data,” the framework implements extensive standards application to maintain accuracy, completeness, and consistency. For “non-target data,” it allows for flexibility to handle variability and contextual differences, maintaining a quality baseline to support CI functionalities. The implementation involves systematic mapping of ML or machine data flows to adhere to data quality protocols, from collection through processing to the formation of a well-curated dataset. Following that, it offers a process guide of five steps for applying the proposed framework to achieve data quality in CI systems, presented in Figure 2 based on the guidelines from the standards. This hybrid approach provides general management strategies to promote quality throughout the entire lifecycle of the data, which can be extended to CI dataset creation and use in complex industrial environments. The process is divided into two main phases. The first phase can be applied before data collection or acquisition and theoretically before system/model development. This phase determines the specification of quality requirements and quality planning, establishing a plan for data quality risk mitigation and management and followed by the second phase of data quality preparation and control. The second phase aims to apply the defined data quality management procedures during data collection and preparation.

To explain further, the first step of the framework is the identification of key quality characteristics, followed by the definition of the associated quality requirements for the global CI system and AI/ML subsystem/s that are affected by the training dataset quality. The quality requirements should be based on the category of the system and subsystem, their function, and criticality, as described in ISO 25030 (ISO/IEC 25030, 2019). These requirements are defined for the system quality in use (QIURs) or directly for the system/product characteristics ((PQRs). QIURs and PQRs requirements and corresponding quality measures can be defined based on the quality model in ISO 25010, ISO 25022 and ISO 25023, respectively. In a second phase, the data quality requirements (DQRs) should be specified directly for the data used by the system/subsystem based on the previously identified system requirements, non-functional requirements and constraints, such as data-domain-specific provisions. In addition to that, DQRs can be defined by the data quality Model in the ISO standard 25012 and quality measures in ISO 25024. The differences of purpose between ML/AI data (target data) and other context data (non-target data) should be taken into account, as well as if their quality impact on the system behavior can be controlled or predicted. This should be considered when setting the strictness of the quality requirements. Afterward, it is important to evaluate the defined quality requirements in order to satisfy them in third step. Prior to data collection and dataset compilation, risks for data quality need to be determined, including the risk associated with human factors, and a method should be specified to mitigate their impact on the data and the system. The three steps above fall within the Requirement Specification and Data Quality Planning stage (first three steps in Figure 2), that outputs a data quality strategy and how to implement it, taking into account the specified data requirements, relevant policies, or domain-related standards. Active management of data quality typically begins only after data collection process (Gong et al., 2023). Once the data quality plan is prepared, the quality implementation procedures for system and human data should be identified, including dataset definition, data acquisition, and quality review as discussed in ISO 22989 as part of the second phase.

In data preparation and data quality monitoring and control, the fourth step in Figure 2 is about addressing and implementing data quality management procedures, following the recommendations associated with the data quality risks identified in the previous step, for each data management stage (a) dataset definition, (b) data acquisition, (c) data annotation, (d) data preparation and cleaning, (e) data augmentation, data sampling, (f) dataset composition, and (g) data quality checking (ISO/IEC 23053, 2022c).The non-target contextual data may not require such management stages, however at minimum, a data quality check should be performed and considered in improvement efforts of the system quality. The fifth step, is about the identification and report of unresolved data non-conformity and quality issues. Potential impacts on the CI system performance should be analysed and quantified when possible and the data quality plan should also be evaluated for further improvement of the processes. This is the final step of the feedback process to systematically improve the data quality and eliminate identified root causes of data non-conformity. This step therefore depends on how data is collected to create a datasets and should guide the final dataset collection.

## 4 Case studies

In this section we demonstrate the application and efficacy of the proposed hybrid standardization approach in managing data quality within two specific case studies in industrial HRI. The case studies were designed and conducted at Irish Manufacturing Research, Ireland. Given that the datasets have not yet been published, we have provided a high level description of the data. We focus on the data quality challenges and management methods used, while offering some practical strategies to ensure good quality CI datasets.

### 4.1 Human-robot teaching interaction through kinesthetic programming by demonstration (PbD)

This case study investigates human and robot teaching interaction using Programming by Demonstration (PbD). PbD is a supervised method which allows humans to impart skill to a robot without explicitly programming the robot through different direct or indirect input methods. One of the direct input methods is kinesthetic teaching, where operators manually guide the robot’s movement to demonstrate specific tasks.

The method is suitable for where traditional programming struggles to adapt complex and frequent customization of tasks in HRI. This approach reduces programming setup time and increases the robot’s adaptability to varied task requirements (Villani et al., 2018).

In developing our case study, we built upon the experimental framework that was detailed in our previous work Mehak et al., 2023). The framework has been specifically designed for collecting multimodal collaborative data. The purpose of collecting this data is three fold; first, to assess human performance and robot’s learning curve in real time as it requires new skills from human demonstration; second, to evaluate the ergonomics and cognitive impact on human operators; and third, to train an ML model to develop an adaptive HRI system. To collect the collaborative data, we used state-of-the-art tools (Lin et al., 2023) and metrics (Skaramagkas et al., 2023) for evaluating human performance and analyzing the robot’s task execution algorithms.

The experimental setup of this study involves a collaborative robotic arm with gravity compensation control to accurately mimic human movements, an RGB-D vision sensor for object tracking, and a mediaPipe-based vision system (Ma et al., 2022) is developed to capture human ergonomic data of teaching postures. We use a wearable eye tracker to capture the cognitive load and focus of human operators during their teaching interactions. During the experiment, operators perform two of predefined tasks, demonstrating each step to the robot. Task one is simple object manipulation and the other task involves object sliding over the slider representing traditional activities on a production line that could benefit from automation. At the end of the experiment, participants are asked to fill out questionnaires to give their subjective feedback. The collected data consists of three sets: human motion data, robot demonstration data and human cognitive data. Further, we applied our hybrid standardization approach and guidelines from data acquisition to dataset compilation, following the procedure illustrated in Figure 2. A comprehensive breakdown of data sources, tasks, and challenges related to data quality that cover the need for high precision and consistency is presented in Table 2.

**TABLE 2:** Use case 1 summarized: data sources, data quality challenges, impact and applicable standards.

Data sources	System operations	Description	Unique challenges in CI	Data quality issues	Impact on system performance	Applicable standards
Human Motion Data	Data collection from vision sensor	Recording of human postures while performing teaching tasks	- Susceptibility to noise, - Variability across individualise	- Issues in data reliability and accuracy	- Impaired evaluation of user performance, - Misalignment leading to inefficient task execution and safety issues	- ISO/IEC 25012 and ISO 800-61
Human cognitive state data	Extracting eye tracking data	Eye-tracking metrics, involving cognitive process and visual attentions recorded during HRI.	- Extensive preprocessing required, - Inconsistent performance due to changes in environmental conditions	Timeliness, and consistence	- Inconsistent performance due to changes in environmental conditions - Delays in data processing can impede the real-time interaction	- ISO/IEC 25012 Data quality model, - ISO 8000-61 Data quality management
Robot data	Data collection from robot sensors	Robot- learned behaviors, which are policies acquired through human demonstrations	- Task adaptation	Accuracy and timeliness	- Reduced system learning efficiency and adaptation efficacy	- ISO/IEC 25012 and ISO/IEC 23053
Unified multimodal data	- Data integration and synchronization	Combined data from different data channels	- Ensuring data coherence across different modalities and managing diverse data sources effectively - Disparities in data types can lead to integration issues	Completeness, integration, and synchronicity	- Incomplete or erroneous responses from the HRI system	—

The following sections detail the implementation of hybrid standardization in our case study.

#### 4.1.1 Quality requirement identification for the system

In this case study, the system consists of two main subsystems; the human subsystem, which is responsible for demonstrating tasks to the robot, and the robot, which learns them and later executes them. The additional subsystems include a human motion tracking system and an eye tracker head system, as previously mentioned. The quality requirements for the motion tracking system are set to capture images at a resolution of 1080p with a frame rate of 60 frames per second, ensuring real-time data processing to accurately track human movements. The eye tracker system is required to operate with a sampling rate of at least 100 Hz and a latency of less than 10 milliseconds to precisely monitor cognitive load and attention. These specific requirements are tailored to the needs of our collaborative robotic training environment, demonstrating that the exact specifications can vary based on the application and system goals. The requirements are identified on the basis of performance efficiency described in (ISO/IEC 25010, 2023a). At the system level, quality requirements focus on ensuring complete data flow and interaction between these subsystems (ISO/IEC 25012, 2008a), scalability to adapt to varying user interactions, and robustness to operate effectively under various situations, as outlined by ISO/IEC 25030 (ISO/IEC 25030, 2019). Some additional requirements are not explicitly covered by these standards, such as real-time adaptation, multimodal data integration, and the scalability necessary for dynamic environmental responsiveness, which are some of the characteristics of collaborative systems (Odong et al., 2022).

#### 4.1.2 Data quality requirements

**FIGURE 3:**
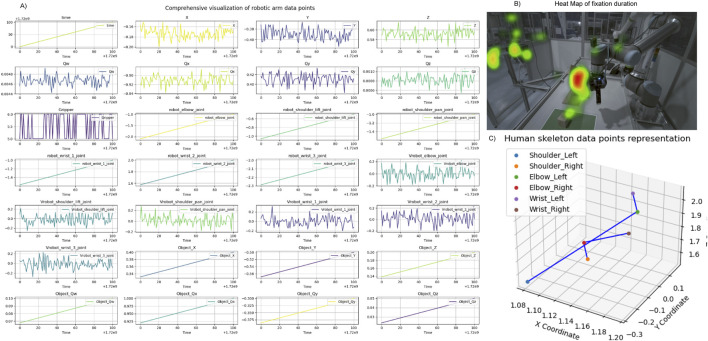
Visualization of data examples from case study 1. **(A)** Display of randomly selected data points derived from signals gathered and processed during the Programming by Demonstration (PbD) task, featuring varying sampling rates and temporal dynamics. **(B)** Heatmap representing the cumulative duration of gaze fixations, superimposed on the robotic setup from a side view. **(C)** Mapping of human posture trajectories, illustrating movement patterns during the interaction.

The quality of data from both human and robotic sources is a key factor in building a reliable HRI system (Mia and Shuford, 2024). This kind of data is often collected in an experimental setting from a diverse group of participants, including some who have never interacted with robots before. The collaborative data for this use case consists of multiple categories of data, including human motion data, robot programming data, user feedback and cognitive aspects of operator recorded during the teaching interaction. The examples of data instances can be visualized in Figure 3. Ensuring the integrity of the data is crucial, as it will be used for system performance assessment and the training of a deep learning model in the future. Therefore, we adhere to ISO/IEC 25012 (ISO/IEC 25012, 2008a) and ISO 8000-61 (ISO 8000-61, 2022a) to define the quality requirements for the data. Data accuracy is one of the important quality requirements for collaborative data (ISO 8000-61, 2022a). In this use case, to ensure accuracy, it is needed to record the actual movement of humans and capture precise positional coordinates, orientation and joint states of the robot. Another quality requirement is defined to ensure data completeness (ISO/IEC 25012, 2008a; ISO 8000-61, 2022a)that no data points are missed and capture a full representation of human and robot interactions. Timeliness is an additional data characteristic (ISO 8000-61, 2022a), with data processed within a 10 m window to provide immediate insights into the operator’s cognitive aspects and integrity throughout the data acquisition phase. These requirements are derived based on the functionalities of each subsystem to maintain data quality.

#### 4.1.3 Risk identification and data quality plan

To identify possible risks related to data acquisition of each type of data we referred to the guidelines presented by data quality (ISO 8000-61, 2022a). The primary risks include accuracy risk, where inaccuracies in task demonstration data could lead to misinterpretation of human actions by the robot resulting in incorrect task execution. Moreover, the completeness risks arise from the vision system’s occlusion problem. This identification highlight that even a minor deviations in recording human postures can lead to substantial errors such as missing data, which in turns can generate inaccuracies in the analysis, thus affecting the overall validity and reliability of the research findings. The risk related to cognitive data involves precisely measuring and timely processing of cognitive assessments such as engagement levels and cognitive loads, which are critical for enabling real-time adaptive teaching responses. Additionally, the subjective nature of user feedback data requires comprehensive design and implementation of feedback methods as they reflect user experiences, thereby supporting accurate system adjustments based on real user feedback.

To manage the defined potential risks in this case study, the data quality measures specified in ISO/IEC 25012 are followed as quality assessment plan.

#### 4.1.4 Quality control in data acquisition

The data acquisition is conducted in a laboratory setting. The participants perform two simple assembly tasks (a) target object placement and (b) object sliding on the slider. While completing the tasks, a head mounted eye tracker system collects participant gaze and pupil dilation. At the same time, the motion tracking system captures the data about their teaching postures which include body’s joint movements. In addition, subjective feedback on workload and usability is also recorded using online questionnaires. These responses are context-driven for developing the understanding of HRI interaction dynamics and refining the robot’s learning algorithms. After data acquisition, an initial investigation of the data was performed to ensure that the data values and formats were accurate, as a data preparation step in (ISO/IEC 23053, 2022c), and the data are categorised into two groups as user data and robot data.

#### 4.1.5 Dataset preparation and quality control management

After data collection, the first step is to extract data from system specific format to user accessible format for data preprocessing involving data cleaning, where inaccuracies and inconsistencies were removed. This involves cleaning out noise from the eye tracking data and motion data, realigning the posture data, and handling missing or extreme values. Furthermore, cleaning of eye tracking data involves eliminating data points with unrealistic values above an acceptable physiological limit for pupil size and eye movement direction. Non-informative spikes such as blinks and data loss due to poor calibration are retained and filled via linear interpolation. Simultaneously, for motion data, the missing values due to occlusions are addressed using smoothing algorithms to impute the data. The data were annotated to allow for contextual meaning that is crucially needed for the preparation of CI data. For instance, annotations pertaining to user data are labeling human postures as “ergonomic” or non-ergonomic and cognitive data that have high task engagement levels inferred from the eye tracking data and questionnaire responses. The next step is to combine both data groups into a complete dataset in order to correlate and analyze data from different sources; this step is called data integration in (ISO/IEC 25012, 2008a). Lastly, the data set is transformed into suitable formats to align with the technical requirements of analysis tools and learning models.

After data set compilation, an internal report was produced to highlight key processes and expert feedback on data quality and unresolved issues. Each data streams present specific issue that were reported with action taken from (ISO 8000-61, 2022a).

### 4.2 Human-in-the-loop telerobotics

**FIGURE 4:**
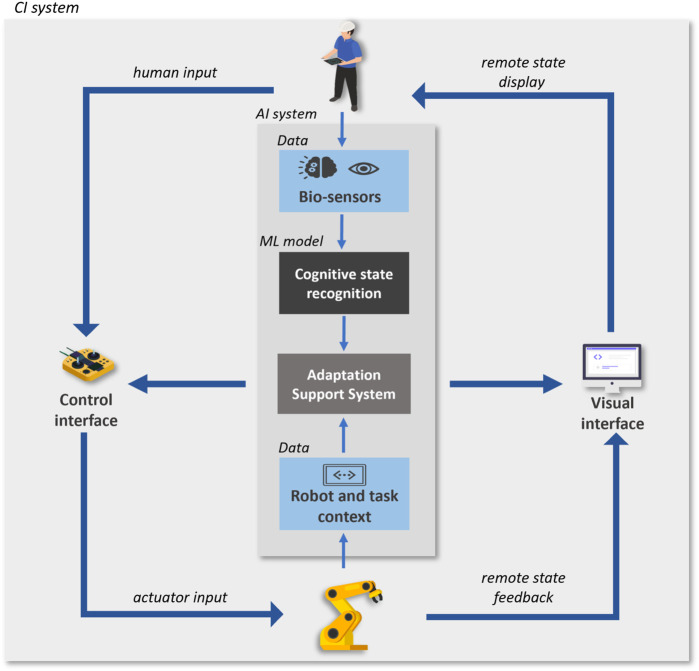
Human-in-the-loop teleoperation CI system with cognitive computing ML component. Icons designed by Freepik, https://www.freepik.com

The human-in-the-loop teleoperation case-study aims to address flexible and dexterous manufacturing tasks by leveraging the strengths of humans and robots (Figure 4). This type of remote operation of a robot is well suited for medical device manufacturing applications, that may involve the use of dangerous tools, maintaining sterile conditions, and manipulation of miniature and malleable device parts. Moreover, it frequently involves high-customization and low-volume production constraints. The human operator bears the important role of adaptability and dexterity, reducing the implementation costs compared to a fully automated system.However, this role can be hindered by the teleoperation interface design when it reduces the operator’s awareness, presence, or engagement in the task. For optimal performance, a human-in-the-loop teleoperation system should be able to monitor and recognize in real-time the operators’ internal state, either cognitive or affective, and adapt accordingly to improve their performance. The system can resort to different adaptation strategies depending on the user needs, such as adjustment of the interface design, changes of the interaction modality or automation level (Dehais et al., 2020). The internal state information can be estimated from different types of human-related data, including behavioural, physical and physiological indicators (Lim et al., 2018). In our CI system, an operator’s mental workload recognition function is implemented by a multimodal end-to-end deep learning model, that fuses two physiological data streams (Electroencephalography (EEG) and Eye-tracking).

An experimental teleoperation setup was developed for data collection, including a remote robot station with a six-degree-of-freedom robotic arm, a hot-wire mounted on the end-effector, and several cameras to provide different views of the remote environment. On the operator station the graphical user interface is presented on a monitor, providing the operator with the real-time camera streams and other robot state information. For training and implementation of the deep-learning model, the physiological data were collected from the subjects and prepared into a dataset. Moreover, data related to the teleoperation interface, robot kinematics, and task performance are recorded simultaneously to provide context, generate labels, or be used as additional input streams to the adaptation mechanism. Next, we specify the key data quality management processes used to achieve the final goal of dataset creation (including dataset definition, data acquisition, data processing and cleaning), particularly those more relevant for this type of CI data and application, guided by the proposed hybrid standardization framework.

#### 4.2.1 System quality requirements

The overarching goal of such a CI system, with collaboration between a human operator and a teleoperation robotic system, is to maintain a clean room environment and enhance productivity in high-customization and low-volume production scenarios. These goals require the system to support robot manipulation dexterity and flexibility, while providing higher handling precision and velocity. The implementation of the CI system includes a computer sub-system, a human collaborator/operator, a robotic sub-system, the sensors sub-systems, the interfaces between them, and the relevant environment for the system. The computer component implements an AI sub-system with the goal of monitoring and real-time recognition of the operators’ internal cognitive state. The operator state recognition function is implemented by a multimodal model that receives as input EEG and Eye-tracking sensor data, and outputs the level of mental workload. The output of the recognition model is communicated to the teleoperation system, which adapts according to the recognized operator state (following a rule-based approach), as depicted in Figure 4. This AI sub-system is not considered safety critical for medical device manufacturing applications, but it is essential to maintain the productivity of the cell.

The first step of the data quality management guide (Figure 2) involves the identification of quality requirements for the system and sub-systems according to their function, criticality, and the context of use. The most relevant quality characteristics of the CI system to meet the stakeholders’ needs in this case study (based on the quality model in (ISO/IEC 25010, 2023a),are performance efficiency, interaction capability, flexibility and reliability. A CI production cell throughput requirement can be set no lower than the manual production throughput, and a performance efficiency measure should be selected to indicate the degree to which the system meets this requirement. As a human-in-the-loop system, the interaction capability measures the degree to which the system is learnable within a specific amount of time, is easy to operate and control, is engaging to the user, and can prevent human error. Considering the high-customization and low-volume production needs, the reliability of the system is important, mainly because it is operational and accessible when required for use, fault tolerant, and flexible to adapt to production parts and production workload variation.

For the AI sub-system instead, functional suitability and compatibility are essential. The sub-system should cover and provide accurate results for all of the specified manufacturing tasks and intended users and should be compatible with the other sub-systems, particularly the sensor systems.

#### 4.2.2 Data quality requirements

To implement the second step of the data quality management guide, we first identified the type of data required for the ML task, then the data quality needs, and the most important quality characteristics to monitor and examine, as advised in ISO 25030 (ISO/IEC 25030, 2019) and ISO 8000-61 (ISO 8000-61, 2022a). The cognitive state recognition component requires synchronized time series signals from biosensors and corresponding human state labels. For this particular use-case, we highlight the importance of accuracy, completeness, consistency, credibility, traceability, and understandability characteristics of the 15 data quality dimensions of the ISO 25012 Data Quality Model (ISO/IEC 25012, 2008a). The accuracy of the training and testing data shall be considered as to how accurately reproduces with high-fidelity the operational data, also required to meet system functional correctness requirements. Some examples of data accuracy measurements are the proportion of outliers in the training dataset compared to the operational data (ISO/IEC 25024, 2015b),the training and operational data distribution difference, or the differences between the acquisition chain used for the development of the model and the one used in operation (Cappi et al., 2021). The compliance with data completeness requirements, and the related data representativeness dimension (Cappi et al., 2021), can be measured by more traditional measures such as the proportion of samples with not null values for a specific attribute/feature (ISO/IEC 25024, 2015b),or more simply as the number of variables and variable values covered by the data from the defined Operational Design Domain (ODD) (Cappi et al., 2021). As the use-case data require extensive pre-processing and the synchronization of multiple data streams, the data collection and processing protocol shall be well annotated and logged, to support credibility and traceability on this high-dimensional data, that is not easily interpreted and understandable by non-experts. Some examples of data credibility measures are the proportion of feature values validated/certified by an expert or specific process (ISO/IEC 25024, 2015b),or the proportion of samples flagged during data collection has potentially affected by a quality degradation source.

#### 4.2.3 Risk identification and data quality plan

Data quality risks were then identified for each of the data quality requirements, and a plan was formulated to prevent or manage the risks, following the third step of the guide. One of the major risks in the dataset definition stage is the non-rigorous definition of the ODD, which does not take into account the manufacturing task to perform or the targeted operational context and consequently introduces bias or collects non-representative samples. A data quality risk specific to this use case is the loss of synchronization between the biosensors, particularly when acquired with different devices and systems. Another major quality risk in CI data acquisition is data contamination by the human operator/agent. Data collection should be tailored towards the target users, e.g., for teleoperation operators, as the skill level of different operators may impact their physiological signals and reactions to different task conditions. Similarly, when the task requires the operator to move, steps should be taken either during data collection or after data collection, to minimize the risk of data quality degradation due to movement artifacts or occlusions. The likelihood and impact of CI data quality issues are task and application dependent and should be estimated accordingly. For example, in industrial tasks carried out inside a building, with fixed lighting levels and interaction through a screen display or virtual reality headset, such as in the present case study, the light effects on the pupil signal and pupil occlusions are less likely than for tasks carried out outside, or for eye tracking data collected with portable glasses. For EEG data, instead, it is empirically known that muscle and movement artifacts have a bigger impact on the signal quality than heart artifacts, due to their magnitude and lower availability of artifact removal/correction methods. Moreover, for multimodal systems, the impact of quality degradation in one of the modalities is expected to be lower than in an unimodal system (Poria et al., 2017). Paradoxically, during data preparation the quality of the data can be affected by data transformations and the cleaning process. It is crucial that the data processing procedure is planned and well justified beforehand, and not changed during or after this stage to avoid the introduction of bias.

Subsequently, we present some practical examples of how these risks were minimized or handled during data acquisition and data preparation. These examples serve as actionable solutions to manage data quality risks.

#### 4.2.4 Quality control management during data acquisition

**FIGURE 5:**
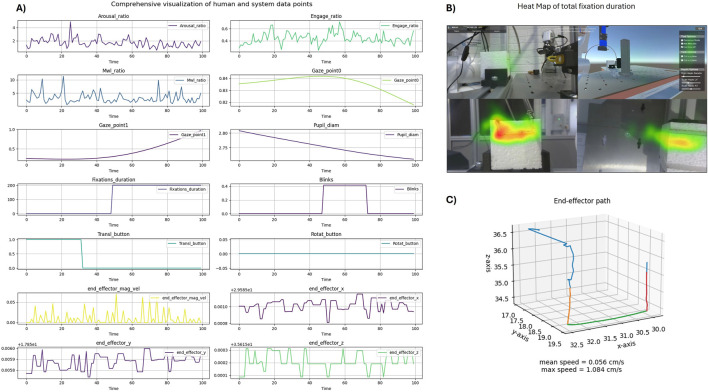
Visualization of data examples from the Human-in-the-loop Telerobotics case-study. **(A)** One hundred data points from signals collected and computed during the teleoperation task, with different sampling rates and temporal dynamics. **(B)** Heat map of the total gaze fixations duration, overlapped on top of the visual interface of the system. **(C)** Robot’s end-effector path during a cutting task.

Having identified the data sources, data features and data quality needs of the system, as part of a dataset definition step (Picard et al., 2020), data collection was planned and carried out in an experimental setting. During the performance of a teleoperation task, a screen-based eye-tracker system recorded the subject’s gaze and pupil changes with a 60 Hz sampling rate, a mobile EEG system recorded the subject’s brain activity changes with a 500 Hz sampling rate, along with simultaneous recording of the robot kinematics, task duration, and performance. After each trial/task, the subject was requested to fill in online questionnaires to assess their perceived cognitive state and to help generate operator state labels for the data. To summarize, three groups of related data were acquired: (a) user state data, (b) user behavior data, and (c) user performance data (see Figure 5 for examples of the data collected).

During data acquisition, data quality monitoring and control procedures were implemented for the identified data quality risks, as advised in ISO 8000-61 (ISO 8000-61, 2022a). The EEG and eye tracking signals were synchronized during collection with the Lab Streaming Layer (LSL) (Kothe et al., 2024), using keyboard events to mark the start and end of the tasks. LSL is a standardized protocol that allows any sensor device that uses it to stream different data types and multiple channels in a real-time, unified, and synchronized way. Using this protocol allowed for easier and higher quality data synchronization; however during data collection, we nevertheless monitored the status of the streams and that the event markers were being recorded (through real-time stream visualization), to catch and correct any synchronization and acquisition errors early. If loss of synchronization still happened during the acquisition, a manual synchronization protocol was implemented post-data collection, albeit having lower precision. This issue was flagged when it happened, and during data preparation, its impact on the data streams was analysed (e.g. lower temporal correlation compared to the other recordings).

For our use case, as the data were collected from non-experts, we monitored the subjects during the task to ensure they did not show signs of confusion about the instructions, as the data recorded in this situation will not be relevant to the ML task. Moreover, we monitored them to detect any potential source of noise or artifacts in the sensor data, coaching the subject to avoid unnecessary movements or moving away from the workstation. This last step is particularly important for the physiological sensors used, as they are sensitive to several subject-related noise and typically have a physical distance limit up to which they can capture the signals. Nonetheless, the collected data should represent the expected movement and range of motion of the human in operational conditions to comply with data representativeness requirements (Cappi et al., 2021). Similarly, for eye-tracking data, special care should be taken to match the expected lighting conditions during operation with those during data collection, when possible to control this factor. When lighting levels are variable, the data can be cleaned during data preparation. However it may not be possible to identify and remove all lighting change effects on the eye-tracking data. Annotations of potential sources of noise and the corresponding time stamps can serve as a preliminary quality report of the data and can aid in the data cleaning and processing step.

The interpretation of CI data, which can include indicators of latent states and partially observable variables, may vary significantly. Therefore, context information should be used for more effective collaborative interactions (Zachary et al., 2015). The user behavioral and performance data was collected and recorded by ROS (a commonly used Robot Operating System), triggered by the same events markers streamed with LSL. The data were added to the final dataset by either synchronizing with the physiological streams (e.g., real-time robot kinematic streams) or by annotating data segments (e.g., annotating the trials with the associated task performance). This context can be used as additional input streams to the model or to label different ML tasks, supporting the reuse of the final dataset to train other human-robot related tasks.

#### 4.2.5 Quality control management during data preparation

Following data acquisition, an initial exploration of the data was performed to confirm that the values and formats are as expected and reasonable for the task - data preparation step in (ISO/IEC 23053, 2022c). Before data cleaning, error rates were computed with respect to NaNs, missing values, invalid data, or outliers, and subsequently, cleaning, filtering, imputation, or discarding of the data was performed according to the acceptable rates (considering how the data streams are segmented for real-time recognition).For example, for a pupil diameter signal with missing data, small gaps can be interpolated over, while long gaps above 40 ms should be marked invalid to avoid introducing unrealistic values (Kret and Sjak-Shie, 2018). The intra- and inter-subject data distribution should be measured to detect inconsistencies within subjects or the population. It should be noted that, for human data, outliers are common and may correspond to a normal variation in the measured signals. We leveraged the data collection annotations and additional context streams to better understand if outlier values were due to normal variability or caused by non-task related sources (e.g. when the subject was distracted or asked for help during the experiment).

The physiological data was further preprocessed and cleaned according to community domain standards. For EEG data, different approaches have been used in the literature (Kastrati et al., 2021). The validity and usefulness of different EEG preprocessing approaches for the use of deep learning are still being studied (Kingphai and Moshfeghi, 2021), as there is a lack of methods to compare them. For our CI application, as fast stream preprocessing is required (within a few seconds for real-time implementation), we employed an automated minimal processing protocol that applies data resampling, spectral filtering, automated artifact correction with the Artifact Subspace Reconstruction algorithm (Mullen et al., 2015), and average re-referencing. This protocol aims to be feasible in online operational conditions and avoids the need for manual intervention, which can introduce bias and discrepancies between the training and operational data acquisition chain. In our use case, movement during the task was limited and eye-related artifacts were the most common type of contamination. Nonetheless, for tasks that require the subjects to move, a minimal processing protocol may not be enough to remove/correct these artifacts. For eye tracking data, the transformation of the signals may first be needed to retrieve data related to the pupil or gaze. The cleaning of these signals should target periods with unrealistic values or distortions due to blinks or other interference with eye tracking. Specifically for pupil-related data, correction for lighting levels may be necessary to increase signal quality and keep only task-related changes (remove pupil light response effects in pupil size) (Lu et al., 2015).

All streams were then segmented into trials according to the keyboard markers and subsequently into a few-second epochs. This segmentation process varies depending on the data acquisition protocol, but it is important to add the relevant metadata so that the link to the raw data is not lost. For our use case, information about the task condition ID, subject ID and epoch order within the trial was kept. As data were collected from different subjects, different days/sessions, and different streams, it is important to normalize and scale it for each subject separately [see (Guo et al., 2022) for examples of the use of normalization methods to reduce subject differences and biases], particularly for subject-independent state recognition models, as advised in (ISO/IEC 23053, 2022c).

#### 4.2.6 Data quality report

After dataset compilation, all the processing steps are reported along with the data, as well as data quality issues that were not resolved or even when corrected may affect the model’s performance. In our use case, we note the identification of synchronization and event marker stream recording errors that were corrected manually, which may introduce variations in the quality of the data. As the effect of these types of errors is not trivial to assess, caution should be taken at the model evaluation stage, estimating the model’s output confidence for the affected data samples (model performance may be over- or under-estimated). Additionally, we recommend the continuous assessment of the suitability of the dataset and production model to the operational data and task. As the interaction technology and the non-linear dynamic nature of human-system interactions evolves in complex CI systems (Karwowski, 2012), so does the risk of concept drift and the quality of the CI solution. An ideal CI system should be able to use real-time dynamic data to retrain, update, and improve its behavior continuously according to the new data.

Table 3 presents a summary of the proposed data quality management processes for the teleoperation case-study.

**TABLE 3:** Use case 2 summarized: data sources, data quality challenges, impact and applicable standards.

Data sources	System operations	Description	Unique challenges in CI	Data quality issues	Impact on system performance	Applicable standards
Data Sources	System Operations	Description	Unique Challenges in CI	Data Quality Issues	Impact on system Performance	Applicable Standards
Human cognitive state data	Sensor data collection	Recording of sensor data as proxy to understand the internal operator state	- High-fidelity reproduction of operational data, - Sample representing well a population, - Data with noise and artifact contamination	Accuracy, Representativeness	Lower generalization ability and recognition performance	ISO/IEC 25012 for Accuracy and Reliability
Human cognitive state data	Sensor data processing	Retrieving meaningful latent information from human sensor data	- Extensive preprocessing required, - Introduction of bias in the processing stage	Credibility, Traceability, and Understandability	Difficulty in tracing and identification of data quality root causes for non-experts on the data type	- ISO/IEC 25012 Data quality model, - ISO 8000-61 Data quality management
Multi-modal data	Data synchronization and integration	Combining heterogeneous data types for analysis and processing	Data coherence across different data types	Data consistency, Integration, Synchronicity	Reduced system learning efficiency and adaptation efficacy	—
Human data, with robot and task context	System real-time adaptation to operator	Adjusting to changes in operator state and performance	Real-time system processing of operator data, with robot and task performance context	Data contextualization in unstructured and complex environment	Reduced system flexibility and efficacy in dynamic conditions	—

## 5 Conclusions and impact for future work

This work was inspired by the empirical challenges that were encountered during data acquisition and management, aimed at developing and testing the application of CI, specifically in the HRI area of the CISC project (Leva and Estrada Lugo, 2023). Some of those challenges can affect the ability of collaborative applications to be fully used in industrial settings, potentially limiting their efficiency.In the HRI context, the difficulty in operationalization of relevant human signals can affect the safety of the human-robot interactions and user experience/ergonomic benefits of HRI, while the quality of human feedback can affect the robot’s knowledge acquisition ability. The presence of noise, unaccounted individual variation and missing data may limit the system’s interaction ability and the implementation of complex system functionalities, such as workspace or workpiece sharing.

Consequently, the article reported on the need for better data integration and data quality management. These issues were generalized, starting with limitations and challenges encountered within the specific case studies. Therefore, it is worth highlighting that they are informed by the technology deployed, the methods used and the software tools available at the time of the experiment. For example, the experimental data collection methods for the telerobotic case study reported in Section 4.2 have several limitations that can have an impact on the quality of the dataset and trained model, namely due to the collection within a controlled lab setting, performing only one specific teleoperation task during data collection, with a limited number of experimental conditions. Even the proprietary software used for EEG and eye tracking data acquisition did not offer a user-friendly protocol for interfacing with each other and did not facilitate the synchronization of the time stamps with other data (no absolute time stamps were recorded). For the case study regarding human-robot teaching interaction in Section 4.1, the real-time feedback from the robot regarding the performance on the quality of the trajectory demonstrated by the user is only observable after an actual execution phase.

The two studies were covered by research ethics approval. However, in general, issues related to the use and monitoring of physiological data, such as those considered in this work, may need to be explored further in connection with the need to monitor human performance and how they can be deployed to avoid possible stigmatization (O’Brolchain and Leva, 2020). Moreover, the impact and relevance of these aspects can be very meaningful, as discussed in Section 2. Following are some important considerations.

• The need for specific human in the loop provisions in safety critical application, required by the EU AI act (Laux et al., 2024), will come in conjunction with the necessity for those applications to demonstrate better integration of their data components to handle the automation/robotic inputs and outputs, the control system inputs and outputs, and the many possible forms of human inputs and outputs such as action, physiological signals and subjective feedback.
• There is a need for improved customization in HRI. The customization relies on utilizing human data for each operator, adjusting interface preferences and task allocation. Successful implementation requires precise, dependable, and timely data for development and training, as poor quality can lead to task inaccuracies and communication disruptions.
• Finally, these CI data quality and management issues are to be supported by other data-related organizational processes, such as the management of multimodal data storage architecture, data transfer, data operations, and security. In addition, organizational structure and human resources are key to implementing and maintaining data quality policies and cultures. These can be subject to other industry standards and guidelines not considered in the present study (which was limited to ISO standards), such as those issued by the International Society of Automation.

### 5.1 Summary of key findings

The key recommendations derived in the present paper can be summarized as follows.

• The use of standardized protocols can ensure seamless integration and processing of multimodal data and address challenges related to data format discrepancies.
• Maintaining robust metadata management practices and documenting the origin, context, and preprocessing history of data can facilitate its traceability and reliability in diverse analytical scenarios.
• Understanding the data types, heterogeneous data sources, nature of data (qualitative or quantitative), and intended use of data within the CI context is of the utmost importance.This understanding provides transparency in system behavior which is useful in ensuring data quality in accordance with established data quality standards.
• CI systems are not immutable and are characterized by their dynamic and adaptive nature, as discussed in both the use cases presented in this paper and the latest literature (Schleiger et al., 2024). We must foster a culture of continuous improvement, where feedback is actively sought and used to refine data processes, improve system performance, and address any emerging issues (such as possible changes in paces and interaction as the level of experience and familiarity between the system and the operator increases over time.

Recent works have started to address data-related issues and their impact on the performance and quality of ML/AI data, mostly focused on mitigating the risk of small, noisy, biased or corrupted datasets through data resampling methods or model optimization for increased robustness to specific quality issues (Whang et al., 2023). The standardization of the data quality management process can help in the systematic prevention of quality degradation, thereby contributing to the improvement of the safety and behavioural performance of data-driven and collaborative systems. Furthermore, with the Artificial Intelligence Act (Laux et al., 2024) coming into force, proposed by the European Commission to regulate AI in the EU, this framework can be used to put a data quality management system in place that ensures compliance with this regulation and supports traceability, accountability and fairness requirements. Finally, taking into consideration the narrow definition of data quality employed in the proposed hybrid standardization framework, that highly depends on the purpose and conditions of the data use, its application to foundation and general purpose AI models is limited. Further work is needed to understand how the lack of a clear link between data purpose and data quality affects these models' quality and trustworthiness, the ethical collection and use of personal data (principle of data minimisation), and the responsible use of data storage resources.

Future research will aim to explore other standards relevant to collaborative systems in other domains, such as automation for the process industry and energy sector related to control rooms and distributed control systems. For prospective collaborative intelligence and decision-support applications, paying attention to aspects similar to those discussed in this paper may also be required.

## Data Availability

The original contributions presented in the study are included in the article; further inquiries can be directed to the corresponding authors.
